# Disseminated Peritoneal Hydatidosis: A Case Report

**DOI:** 10.31729/jnma.9113

**Published:** 2025-06-30

**Authors:** Shashi Yadav, Prajwal Neupane, Kunal Pathak, Amit Bhattarai, Ujjwal Kumar Jha

**Affiliations:** 1 Patan Academy of Health Sciences, Lagankhel, Lalitpur, Nepal; 2 Nobel Medical College, Biratnagar, Morang, Nepal

**Keywords:** *albendazole*, *disseminated peritoneal hydatidosis*, *hydatid cyst*, *laparotomy*

## Abstract

Hydatid disease or echinococcosis is caused by a tapeworm of the genus Echinococcus. Several cases of hepatic hydatid cysts are reported worldwide, but disseminated peritoneal hydatidosis with spontaneous rupture of cysts leading to ascites is a rare entity, and only a few instances in literature can be found. We would like to report case of disseminated peritoneal hydatidosis, which was secondary to the rupture of the primary hepatic hydatid cyst. Midline laparotomy with partial pericystectomy with removal of daughter cyst and peritoneal lavage was done.

## INTRODUCTION

The echinococcal infestation is due to accidental ingestion of echinococcal eggs passed from canine (definitive host) feces, which are ingested incidentally by cattle (intermediate host).^1,2^ Most of the cases are seen in cattle-grazing countries and countries where animal husbandry is common.^3^ Hydatid cysts are spherical, fluid-filled, unilocular vesicles that consist of an internal cellular layer (germinal layer) and an outer acellular layer (laminated layer). The gradual expansion of the parasitic cyst results in a granulomatous host reaction, followed by the development of a fibrous layer known as pericyst.^4^ The most commonly affected organs are the liver, lungs, brain, bones, kidneys, and heart. HD is usually asymptomatic but can cause morbidity and mortality. It is a life-threatening zoonotic disease, recognized as a major public health problem worldwide.^5^

Hydatid disease is found all over the globe and has been a burden for so many peoples. Rupture of cysts with peritoneal dissemination is a life threatening that needs immediate surgical attention. We believe reading this paper after its publication, it aids operating surgeon to manage cases of hydatid cysts with peritoneal dissemination more efficiently.

## CASE REPORT

An eighty-five-year-old female presented in the surgery outpatient department with a complaint of abdominal pain and mild swelling of the bilateral lower limb for one month. The pain was localized in the right upper quadrant over a swelling , which was insidious in onset, mild, dull aching, associated with loss of appetite for one week with no aggravating or relieving factor. The swelling on the right upper abdomen has gradually increased in size over thirty years. She has no history of fever, loose motion, constipation, shortness of breath, chronic cough, palpitations, and heavy weight lifting. There was no history of trauma, weight loss and similar swelling elsewhere in the body. She has no history of keeping pets.

On physical examination, pallor, icterus, lymphadenopathy, clubbing, and dehydration were absent. There was mild bilateral pitting edema of the lower limbs. A prominent bulge in the right upper quadrant was noticed in the standing position. The swelling was palpable, spherical in shape, around 6*6 cm square in size, mobile in one direction, smooth, non-tender on palpation, with no pulsatility, with smooth margin and was also present while coughing. It was reduced on sleeping at the left lateral position. Ultrasound of the abdomen and pelvis revealed liver cysts with ascites and intraperitoneal hydatid cysts. CT scan was done to evaluate the size, location, state of surrounding structures, the number of abdominal hydatid cysts and to plan for operation. Hematological, biochemical, and radiological tests were performed. Hb 11.4gm/dL, WBC count 14600/mm^3^, neutrophil 90%, lymphocytes 4%. Other hematological and biochemical tests were within normal limits. An ELISA test to detect antibody titer against echinococcus antigen was not performed as it was not available at the center.

The presence of ascites in ultrasonography and a CT scan of the abdomen confirmed the cysts to be ruptured, for which an emergency midline laparotomy was performed. Initial intraoperative findings included multiple intraperitoneal cysts floating in ascitic fluid, which were noted and removed. The omentum was thickened and rolled, and a few cysts adhered to the surface of the liver. A peri cystectomy was done to remove hepatic cysts. Partial peri cystectomy was performed for hepatic cysts that were ruptured and tightly adhered to the liver parenchyma. After the removal of all the cysts, peritoneal lavage with 10% betadine was done. Two drains, one in the subhepatic and the other in the pelvis, were placed. No signs of anaphylaxis were noted intraoperatively, and the patient was extubated after the operation. Few cysts were sent for histopathological evaluation where multiple sections examined from the specimen showed laminated, acellular layer and hyaline ectocyst layer as well as innermost nucleated endocyst/germinal layer with numerous daughter cysts and brood capsules with scolilices. Hydatid sand was also noted. Pre and post-operative prophylactic antibiotics along with an antihelminthic agent (albendazole) were prescribed.

**Figure 1:**
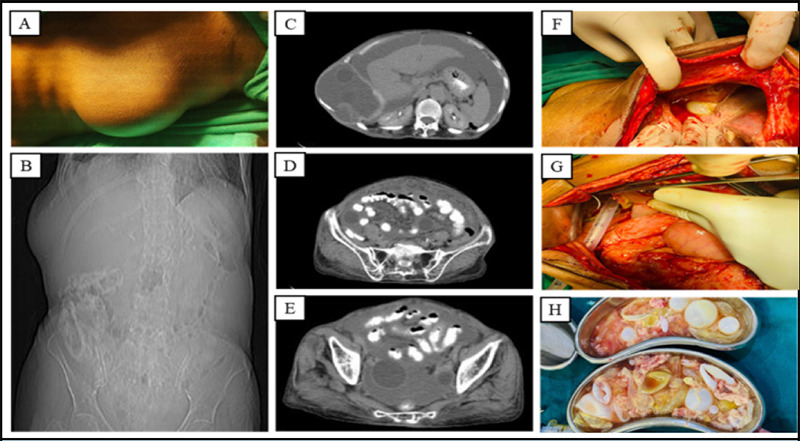
(A) Prominent bulge in the right upper abdomen. (B) X-ray of the abdomen and pelvis showing the bulge. (C-E) CT scan of the abdomen and pelvis shows multiple cysts with ascites. (F) Ascites in subhepatic region. (G) Intraoperative drain placement. (H) Postoperative surgical specimen of multiple hydatid cysts.

## DISCUSSION

Hydatid Disease (HD) or echinococcosis is a zoonotic disease caused by tapeworms: E. multilocularis, E. granulosus, E. oligarthrus, and E. vogelis. The commonest causes of human alveolar and cystic HDs are E. multilocularis and E. granulosus, respectively.^12^ Ingestion of echinococcal eggs from either canines or grazing cattle is the cause of transmission into humans. The liver is the most commonly affected organ, with an infestation rate of 60-75%. Specifically, the right hepatic lobe is affected in 80% of cases and the left lobe in 20%. Other less common sites include the lungs (15%), spleen, peritoneum, kidneys, and the brain.^6,7^ Peritoneal hydatidosis is an uncommon presentation of all HDs. Primary peritoneal hydatidosis is a rare entity and accounts for only 2% of cases of peritoneal hydatidosis.^7^ However, disseminated peritoneal hydatidosis is a common presentation of peritoneal hydatidosis and is a result of implantation due to surgical, traumatic, or spontaneous rupture. Nearly 12% of cases of peritoneal HDs are due to spontaneous rupture of hepatic, splenic, or mesenteric cysts^8^. Some small cysts may eventually become calcified. Spontaneous intraperitoneal rupture of a hepatic hydatid cyst can lead to ascites rarely.^9^ Many cases of HDs are asymptomatic for years before presenting with abdominal pain, which is due to the pressure effect of the cyst. Expansion of the cyst wall eventually leads to rupture, resulting in dissemination and pyogenic bacterial infection that increases the morbidity and mortality.^10^ Rupture of the cyst relieves abdominal pain and makes the patient feel more comfortable.^10,11^ Other abdominal symptoms are nonspecific, like abdominal fullness, dyspepsia, anorexia, and vomiting.^7^

Clinical presentation of swelling may initially lead to confusion and misdiagnosis as a hernia. The absence of definitive radiological evidence of hydatidosis in the liver, lungs, or abdomen raises concerns, particularly given the rarity of ovarian hydatidosis. This condition is often misidentified as a malignant cystic ovarian tumor, which can result in unnecessary surgical interventions.^12^ Anti-helminthics like albendazole or mebendazole are the mainstay of treatment for uncomplicated cases. Surgery, along with pre and postanthelmintic therapy, is indicated for disseminated cases. Combination therapy with albendazole and praziquantel is effective in the treatment of hydatid cysts and can be used as an alternative to surgery in disseminated and non-operable cases.^13^ The minimally invasive Puncture Aspiration Injection Reaspiration (PAIR) with scolicidal agents like hydrogen peroxide, betadine, hypertonic saline, chlorhexidine, and cetrimide is available for uncomplicated cysts that do not respond to initial medical therapy and can be assessed percutaneously.^14,15^ Peritoneal lavage with scolicidal agents is performed in cases with rupture. Spontaneous or intraoperative rupture of cyst is common in 20-50% of cases and may present with symptoms and signs of allergy, anaphylaxis, and peritoneal irritation, and in 5-8% of cases with secondary bacterial infection.^16-18^

## CONCLUSIONS

The treatment strategy involving open midline laparotomy, removal of an intraperitoneal daughter cyst, and partial pericystectomy for a ruptured hepatic cyst that was tightly adhered to the liver parenchyma, along with peritoneal lavage using 10% Betadine and post-operative albendazole therapy, was the preferred method in this case. The risk of echinococcus should be considered where cattle rearing occurs near human dwellings. Visual inspection of the abdomen may not provide an accurate assessment. The typical approach for diagnosing hydatid disease and peritoneal dissemination involves ultrasonography followed by a CT scan, which are commonly utilized imaging modalities.

## References

[1] Gessese AT (2020). Review on Epidemiology and Public Health Significance of Hydatidosis.. Veterinary Medicine International..

[2] Eckert J, Deplazes P (2004). Biological, Epidemiological, and Clinical Aspects of Echinococcosis, a Zoonosis of Increasing Concern.. Clinical Microbiology Reviews..

[3] Grosso G (2012). Worldwide Epidemiology of Liver Hydatidosis Including the Mediterranean Area.. World Journal of Gastroenterology..

[4] Nunnari G (2012). Hepatic Echinococcosis: Clinical and Therapeutic Aspects.. World Journal of Gastroenterology..

[5] Hammami A (2015). Unusual Presentation of Severely Disseminated and Rapidly Progressive Hydatic Cyst: Malignant Hydatidosis.. World Journal of Hepatology..

[6] Beggs I (1985). The Radiology of Hydatid Disease.. American Journal of Roentgenology..

[7] Hegde N, Hiremath B (2013). Primary Peritoneal Hydatidosis.. BMJ Case Reports..

[8] Yuksel M, Demirpolat G, Sever A, Bakaris S, Bulbuloglu E, Elmas N (2007). Hydatid Disease Involving Some Rare Locations in the Body: A Pictorial Essay.. Korean Journal of Radiology..

[9] Limeme M, Yahyaoui S, Zaghouani H (2014). Spontaneous Intraperitoneal Rupture of Hepatic Hydatid Cyst: A Rare Cause of Ascites.. BMC Surgery..

[10] Karakaya K (2007). Spontaneous Rupture of a Hepatic Hydatid Cyst into the Peritoneum Causing Only Mild Abdominal Pain: A Case Report.. World Journal of Gastroenterology..

[11] Erdogmus B, Yazici B, Akcan Y, Ozdere BA, Korkmaz U, Alcelik A (2005). Latent Fatality Due to Hydatid Cyst Rupture After a Severe Cough Episode.. The Tohoku Journal of Experimental Medicine..

[12] Sing P, Mushtaq D, Verma N, Mahajan NC (2010). Pelvic Hydatidosis Mimicking a Malignant Multicystic Ovarian Tumor.. The Korean Journal of Parasitology..

[13] Jamshidi M, Mohraz M, Zangeneh M, Jamshidi A (2008). The Effect of Combination Therapy With Albendazole and Praziquantel on Hydatid Cyst Treatment.. Parasitology Research..

[14] Anadol D, zelik U, Kiper N, Gmen A (2001). Treatment of Hydatid Disease.. Paediatric Drugs..

[15] Mouaqit O, Hibatallah A, Oussaden A, Maazaz K, Taleb KA (2013). Acute Intraperitoneal Rupture of Hydatid Cysts: A Surgical Experience With 14 Cases.. World Journal of Emergency Surgery..

[16] Derici H, Tansug T, Reyhan E, Bozdag AD, Nazli O (2006). Acute Intraperitoneal Rupture of Hydatid Cysts.. World Journal of Surgery..

[17] de Diego Choliz J, Lecumberri Olaverri F, Franquet Casas T, Ostiz Zubieta S (1982). Computed Tomography in Hepatic Echinococcosis.. American Journal of Roentgenology..

[18] Alexiou K, Mitsos S, Fotopoulos A, Karanikas I, Tavernaraki K, Konstantinidis F (2012). Complications of Hydatid Cysts of the Liver: Spiral Computed Tomography Findings.. Gastroenterology Research.

